# Coexpression of gene Oct4 and Nanog initiates stem cell characteristics in hepatocellular carcinoma and promotes epithelial-mesenchymal transition through activation of Stat3/Snail signaling

**DOI:** 10.1186/s13045-015-0119-3

**Published:** 2015-03-11

**Authors:** Xin Yin, Bo-Heng Zhang, Su-Su Zheng, Dong-Mei Gao, Shuang-Jian Qiu, Wei-Zhong Wu, Zheng-Gang Ren

**Affiliations:** Liver Cancer Institute, Zhong Shan Hospital and Key Laboratory of Carcinogenesis and Cancer Invasion, Ministry of Education, Fudan University, 136 Yi Xue Yuan Road, Shanghai, 200032 China

**Keywords:** Hepatocellular carcinoma, Oct4, Nanog, Stat3, Epithelial-mesenchymal transition

## Abstract

**Background:**

Oct4 and Nanog are key regulatory genes that maintain the pluripotency and self-renewal properties of embryonic stem cells. We previously reported that the two stemness markers were tightly associated with cancer progression and poor outcomes of hepatocellular carcinoma. In this study, we demonstrate that coexpression of Oct4/Nanog modulates activation of signal transducer and activator of transcription 3 (Stat3), an oncogenic transcription factor that is activated in many human malignancies including hepatocellular carcinoma (HCC), as well as the expression of Snail, a key regulator implicated in epithelial-mesenchymal transition and tumor metastasis.

**Methods:**

Oct4 and Nanog were ectopic expressed in MHCC97-L cell lines via lentiviral gene transfection. The stemness characteristics including self-renewal, proliferation, chemoresistance, and tumorigenicity were assessed. The effect of coexpression of Oct4 and Nanog on epithelial-mesenchymal transition change, and the underlying molecular signaling was investigated.

**Results:**

Ectopic coexpression of Oct4 and Nanog empowered MHCC97-L cells with cancer stem cell (CSC) properties, including self-renewal, extensive proliferation, drug resistance, and high tumorigenic capacity. Significantly, Oct4 and Nanog encouraged epithelial-mesenchymal transition change contributing to tumor migration, invasion/metastasis *in vitro* and *in vivo*. Following molecular mechanism investigation indicated Oct4/Nanog-regulated epithelial-mesenchymal transition change through Stat3-dependent Snail activation. Moreover, silencing Stat3 abrogates Oct4/Nanog-mediated epithelial-mesenchymal transition (EMT) change and invasion/metastasis in HCC.

**Conclusions:**

We delineate Oct4 and Nanog initiate stem cell characteristics in hepatocellular carcinoma and promote epithelial-mesenchymal transition through activation of Stat3/Snail signaling. Our findings propose Stat3/Snail pathway as a novel therapeutic target for the treatment of progression and metastasis of HCC with CSC-like signatures and epithelial-mesenchymal transition phenotype.

**Electronic supplementary material:**

The online version of this article (doi:10.1186/s13045-015-0119-3) contains supplementary material, which is available to authorized users.

## Background

Hepatocellular carcinoma (HCC) is one of the most malignant tumors worldwide, and the majority of HCC-related deaths occur due to frequent intrahepatic spread and extrahepatic metastasis [1]. Despite enormous progress has been made in the treatment of HCC, the mortality rate remains high. A better understanding of the biology of the tumors would have a major impact on the management of the disease.

The major advance of tumor biology in recent years has been the discovery of the cancer stem cells (CSCs). CSCs are reported to have inherently greater tumor-initiating potential, which is implicated in tumor relapse, driving primary tumor growth as well as the seeding and establishment of metastases [2]. To date, the existence of CSCs has been demonstrated in a wide variety of malignancies including breast cancer, leukemia, glioblastoma, liver carcinoma, and so on [3-6]. CSCs theory opens up new possibilities of generating novel targets, diminishing resistance to chemoradiation, improving therapeutic efficacy, and improving patient outcome.

It is generally proposed that cancer stem cells originate either from adult stem cells that have lost control of proliferation, or progenitor cells that have acquired the ability to self-renew. However, several studies seem to support the theory that CSCs arise from differentiated tumor cells that have undergone a process of dedifferentiation to become more stem-like. For example, it has been demonstrated that differentiated breast cancer cells that underwent epithelial-mesenchymal transition (EMT) were found to exhibit a more CSC-like phenotype [7]. Evidence from other studies also indicated that the CSC-like cells might be generated with processes that are related to activation of the EMT, which impacts cell differentiation and tumor metastatic potential [8]. Thus, CSC biology and the EMT are thought to be mechanistically correlated and may be key components of cancer progression and metastasis [7]. An in-depth investigation of crosstalk of cancer stemness with EMT is essential to a better understanding of tumor progression from a stem cell model perspective.

Oct4 and Nanog are transcription factors essential for maintaining stem cell phenotypes. Oct4, a homeobox-containing transcription factor, was originally shown to be one of the essential factors regulating pluripotency and self-renewal properties in embryonic stem cells [9]. Nanog, a downstream target of Oct4, expressed in embryonic pluripotent stem cells, contributes to cell fate determination of the pluripotent inner cell mass during embryonic development [10]. Several lines of evidence have suggested that expression of Oct4 and Nanog is closely related to tumorigenesis, tumor metastasis, and distant recurrence after treatment [11,12]. In our previous study [13], we have identified that expression of Oct4 and Nanog are highly related to metastatic potential of HCC cells. Moreover, coexpression of Oct4 and Nanog is a strong independent predictor of tumor recurrence and unfavorable outcome in HCC patients. Based on these findings, CSC theory as well as EMT program, we propose a hypothesis that overexpression of stem cell markers Oct4 and Nanog, which maintain self-renewal and proliferation in embryonic stem cells, may also maintain HCC cell self-renewal, proliferation, metastasis through initiating CSC-like properties, and promoting EMT. To this end, we investigate the role of coexpression of Oct4 and Nanog in CSC-like traits, EMT, and metastasis in HCC. We illustrate that coexpression of Oct4 and Nanog initiates stem cell characteristics in HCC and promotes epithelial-mesenchymal transition through activation of Stat3/Snail signaling.

## Results

### Coexpression Oct4 and Nanog enhances cancer stem-like properties in HCC cells

**Figure 1:**
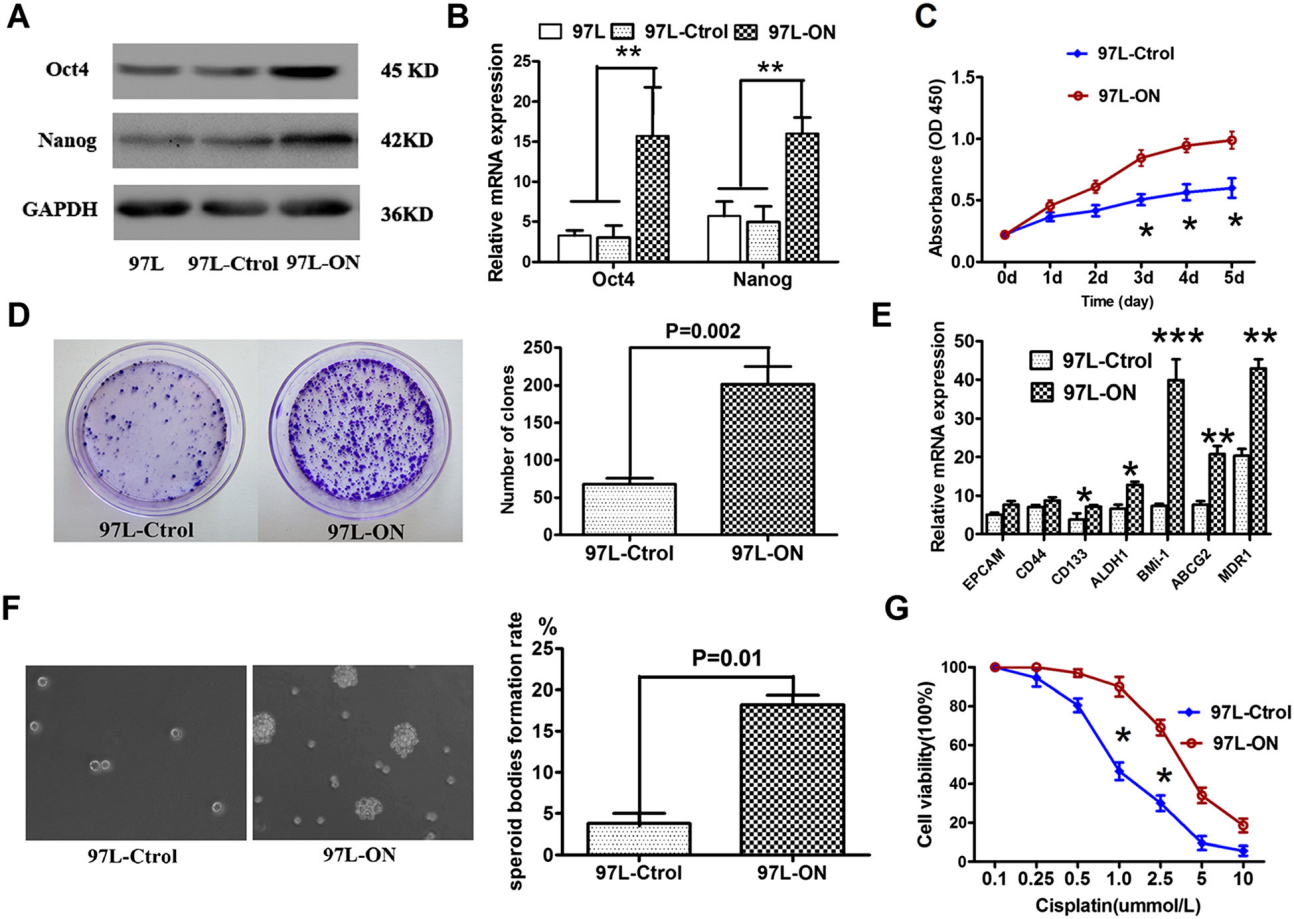
**Oct4**/**Nanog overexpression enhances cancer stem**-**like property and resistance ability in 97 L cell line. (A,** **B)** MHCC97L cells were infected with lentiviral vectors encoding cDNA of Oct4 and Nanog (97 L-ON) or a control empty vector (97 L-Ctrl). 97 L-ON cells were analyzed by Western blot and real-time RT-PCR for Oct4 and Nanog expression (*P < 0.05, **P < 0.01). **(C)** Cell proliferation of 97 L-ON and 97 L-Ctrol cells by CCK-8 assay analysis (*P < 0.05). Proliferation is significant promoted in 97 L cell lines after overexpression of Oct4 and Nanog genes. **(D)** 97 L-ON cells and 97 L-Ctrl cells were subjected to sphere formation assay. The sphere formation was photographed (left) and quantified (right). Colony formation of 97 L-ON cells was increased significantly in comparison with 97 L-Ctrol cells (68 ± 19 vs. 202 ± 38, *P* = 0.002). **(E)** The mRNA expression of stem cell markers (CD133, CD44, ALDH1, BMi-1, ABCG2, MDR1) between 97 L-ON cells and 97 L-Ctrol cells was analyzed by real-time RT-PCR, *P < 0.05, **P < 0.01, ***P < 0.001 **(F)** 97 L-ON cells formed the self-renewing spheroid bodies (left). The spheroid body formation rate of 97 L-ON cells was higher than that of 97 L-Ctrol cells (right, 18 ± 3 vs. 4 ± 1, P = 0.01). **(G)** 97 L-ON cells were treated with cisplatin (0.1, 0.25, 0.5, 1.0, 2.5, 5, 10 μmol/L) for 48 h. Cell survival was determined by CCK-8 assay (*P < 0.05).

To define the role of Oct4 and Nanog in stem-like properties, we generated stable cell lines (97 L-ON) from 97 L human HCC cells using lentiviral infection system with plasmid vectors encoding Oct4 and Nanog cDNA. An empty vector-transfected control (97 L-Ctrl) was produced simultaneously. The exogenously expressed Oct4 and Nanog in HCC stable clones were confirmed by Western blot (Figure 1A) and quantitative real-time PCR (Figure 1B).

We examined the expression of stem cell markers in our 97 L-ON cells. Quantitative PCR analysis showed markedly elevated expression of stem cell-associated genes, including CD133, BMi-1, and aldehyde dehydrogenase 1 (ALDH1), which were found in 97 L-ON cells (Figure 1E) but not in 97 L-control cells.

Given that self-renewal is a hallmark of CSCs, we performed colony formation ability assay and sphere-forming ability assay to investigate the role of Oct4 and Nanog in promoting self-renewal property in HCC cells. As shown in Figure 1D, 97 L-ON cells possessed an increased ability to form colonies after culture in comparison with the control group (mean colony numbers: 68 ± 19 vs. 202 ± 38, *P* = 0.002). In sphere-forming ability assay, few non-adherent spheres were observable in 97 L-ON cells after *in vitro* culture for 1 week and these continued to expand for 2 to 3 weeks in serum-free media. Significant difference was found in speroid body formation between 97 L-Ctrol cells and 97 L-ON cells (Figure 1F, 4 ± 1 vs. 18 ± 3, *P* = 0.01). These results indicated that coexpression of Oct4 and Nanog promoted the tumorigenicity of HCC cells by enhancing their self-renewal abilities.

Resistance to chemotherapy is another important characteristic of CSCs. The 97 L-ON cells displayed increased resistance to cisplatin (DDP) than 97 L-control cells (Figure 1G). Quantitative real-time PCR also showed that the ABC family of multidrug resistant genes ABCG2 and MDR1 were highly enhanced in 97 L-ON cell (Figure 1E).

### Coexpression of Oct4 and Nanog promotes EMT changes and increases HCC cell proliferation, invasion, and metastasis *in vitro*

**Figure 2:**
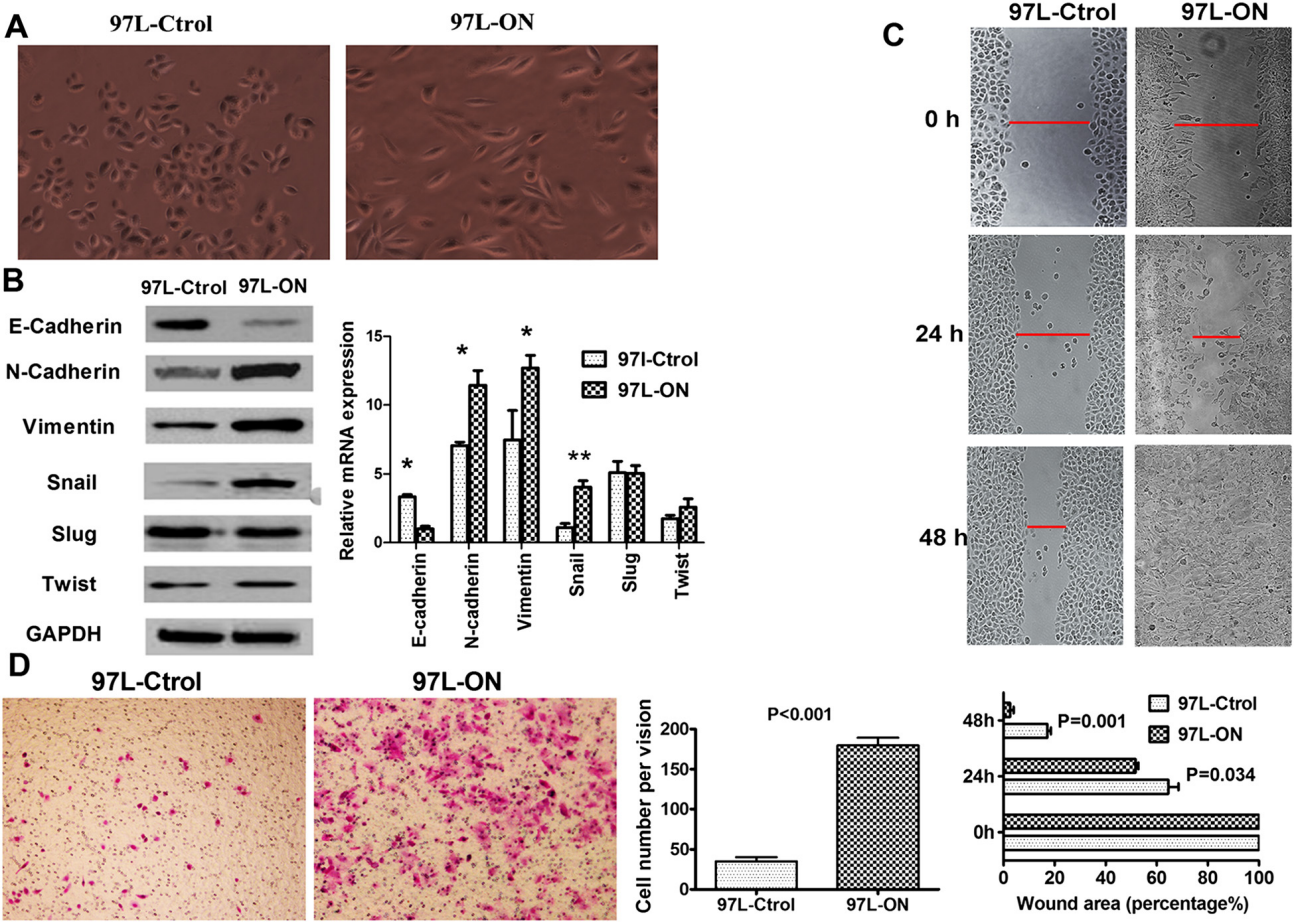
**Overexpression of Oct4 and Nanog promotes EMT and proliferation, migration, and invasion in HCC Cells. (A)** 97 L cells undergo dramatic morphologic changes into mesenchymal, fibroblast-like phenotype after treatment with ectopic Oct4 and Nanog expressions. **(B)** Alterations in EMT genes with overexpression of Oct4 and Nanog. Quantitative RT-PCR and immunoblot analysis of epithelial and mesenchymal mRNA and proteins expression in 97 L-ON cells and 97 L-Ctrol cells. Significant increases in the mesenchymal genes, N-cadherin, Snail, and Vimentin and decrease in the epithelial gene E-Cadherin were found in 97 L-ON cells at both mRNA and protein levels (* *P* < 0.05,** *P* < 0.01). **(C)** The wound healing assay showed that the closure of 97 L-Ctrol cells were significantly slower than that of 97 L-ON cells (*P* = 0.001). **(D)** In transwell assay, cells that invaded through the Matrigel-coated inserts were stained, counted, and photographed under a light microscope at × 100 magnification. Quantification invasion assay indicated that the number of 97 L-ON cells that passed through the Matrigel was more than that of 97 L-Ctrol cells (179 ± 23 vs. 35 ± 13, *P* < 0.001).

After exogenously expressing Oct4 and Nanog in 97 L cells, a typical morphological change was noted in 97 L-ON cells. The cellular morphology was converted to a spindle-shaped, fibroblast-like morphology, as compared with control cell (Figure 2A). This phenomenon suggested that Oct4 and Nanog were involved in the EMT of HCC. We further investigated putative EMT-related markers, such as E/N-cadherin, Vimentin, Snail, Slug, and Twist by real-time PCR and Western blot. The results revealed that overexpression of Oct4 and Nanog led to a significant increase in the mesenchymal genes, N-cadherin, Snail, and Vimentin and a decrease in the epithelial gene E-Cadherin at both mRNA and protein levels (Figure 2B). These observations indicated that coexpression of Oct4 and Nanog could induce the EMT of HCC.

Since the acquisition of mesenchymal properties is associated with increased migratory and invasive properties, we investigated the effect of Oct4 and Nanog on HCC proliferation, invasion, and metastasis. As showed in Figure 1C, CCK-8 assay revealed that cell proliferation was significantly promoted by coexpression of Oct4 and Nanog (*P* < 0.001). We further determined the roles of Oct4 and Nanog in HCC migration and invasion. Wound healing assay showed that the closure of 97 L-Ctrol cells was significantly slower than that of 97 L-ON cells (Figure 2C, *P* = 0.001). Transwell invasion assay indicated that the number of 97 L-ON cells that passed through the Matrigel was much more than that of 97 L-Ctrol cells (179 ± 23 vs. 35 ± 13, *P* < 0.001, Figure 2D). These results showed that endogenous expression of Oct4 and Nanog was associated with HCC invasion and metastasis *in vitro*.

### Coexpression of Oct4 and Nanog promotes tumorigenic and metastatic abilities *in vivo*

**Figure 3:**
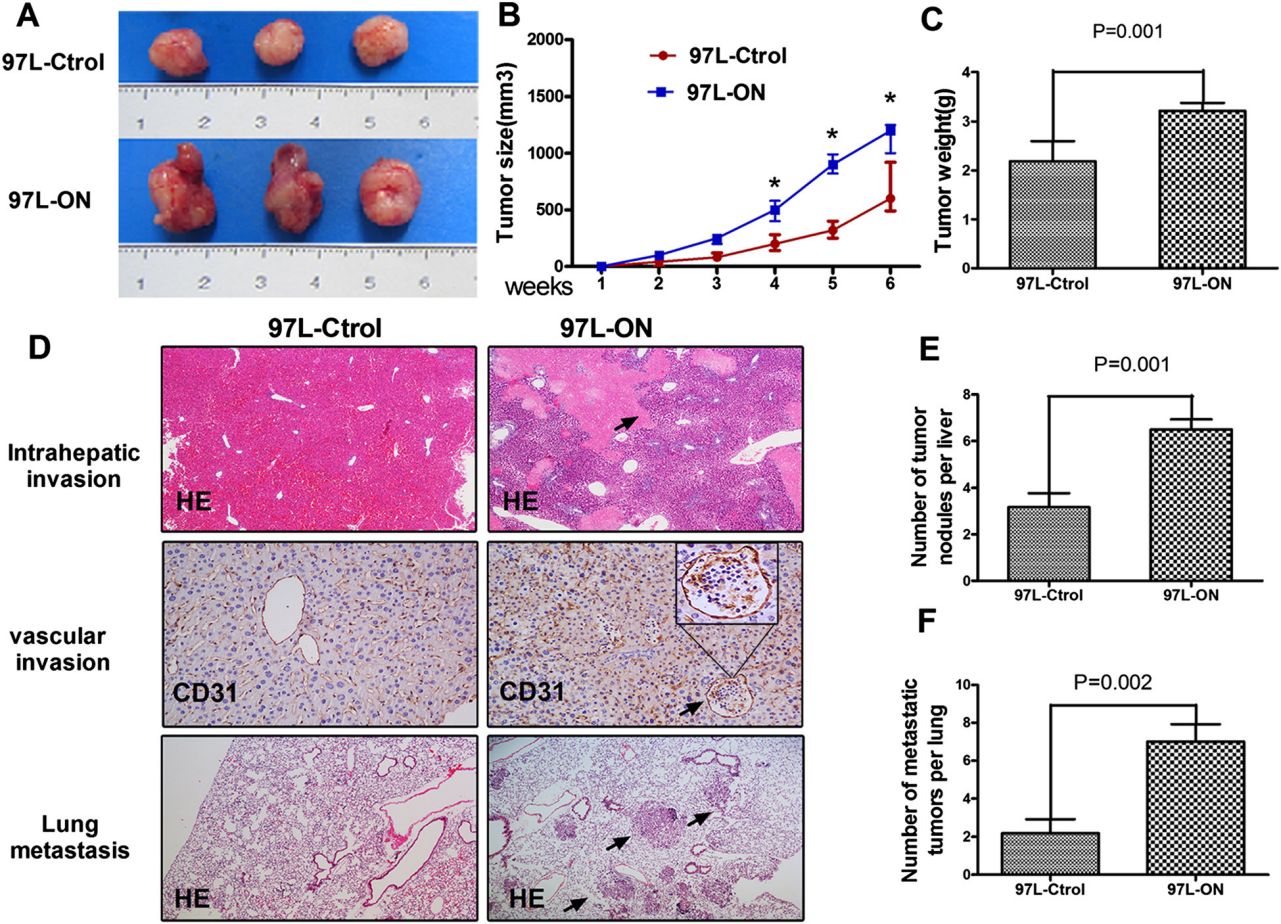
**Overexpression Oct4 and Nanog promotes proliferation and metastasis of HCC in vivo. (A)** 97 L-Ctrol cells and 97 L-ON cells were injected subcutaneously into the right flank of nude mice. Six weeks after implantation, 97 L-ON cells produced larger tumors than control cells. **(B)** The tumor size from each group was measured every 3 days and weighed before dissection. There are significant differences in tumor size between the two groups (all *P* < 0.05). **(C)** The average weight of the 97 L-ON tumors was significantly increased compared with the 97 L-Ctrol tumors (*P* = 0.001). **(D)** The characteristic pictures of intrahepatic invasion (top, H & E staining, black arrows point to the invasive nodules), vascular invasion (middle, CD31 staining, black arrows point to the vascular invasion), and lung metastasis (bottom, H & E, black arrows point to the metastatic nodules in lung). **(E)** Number of disseminated tumor nodules in liver. More intrahepatic disseminated tumor nodules can be found in the 97 L-ON group (*P* = 0.03). **(F)** Number of metastatic tumor nodules in lung. More metastatic nodules in lung can be found in the 97 L-ON group (*P* = 0.018).

Based on the *in vitro* findings described above, we examined the effect of Oct4 and Nanog on tumor growth and metastasis *in vivo*. Xenografts in nude mice were established by subcutaneous injection of 97 L-ON cells and 97 L-Ctrol cells into nude mice as described in the Methods section. Tumor size was monitored every 3 days with a caliper. The tumor growth of the xenografts derived from the 97 L-ON cells was comparable to that of the 97 L-Ctrol cells, showing a marked increase in tumor volume (*P* < 0.05, Figure 3A, B). In addition, the final mean tumor weight of the 97 L-ON group was significantly heavier than that of the 97 L-Ctrol group (*P* = 0.001, Figure 3C), which indicated that the overexpression of Oct4 and Nanog promoted growth of 97 L cells *in vivo*.

A serial dilution experiment was performed to evaluate the *in vivo* tumorigenecity of 97 L-ON and 97 L-Ctrol cells. Nude mice were injected with different number of cells as indicated. 97 L-ON, but not 97 L-Ctrol, generated tumors with the cell number as low as 5 × 10^3^ cells (Table 1).

**Table 1:** **In vivo serial tumorigenicity experiments of 97 L-Ctrol cells and 97 L-ON cells**

**Cell type**	**Cell number injected**	**Tumor incidence**	**Latency (days)**
97 L-Ctrol	5*10^3^	0/6	NA
	5*10^4^	0/6	NA
	5*10^5^	1/6	24
97 L-ON	5*10^3^	1/6	22
	5*10^4^	2/6	14 ± 3
	5*10^5^	4/6	12 ± 2

N/A: results not available.

To address the effects of Oct4/Nanog on HCC cells invasion and metastasis *in vivo*, we established an orthotopic xenograft model in nude mice. After 42 days, 97 L-ON xenograft tumors displayed conspicuous intrahepatic invasion and vascular invasion, compared with 97 L-Ctrol cells (Figure 3D). Then, the incidences of liver dissemination and lung metastasis in the 97 L-ON model and 97 L-Ctrol model were evaluated. As expected, the numbers of metastatic foci in liver (Figure 3E, *P* = 0.001) and lung (Figure 3F, *P* = 0.002) of recipient mice were significantly increased by ectopic overexpression of Oct4/Nanog. Together, these findings demonstrated coexpression of Oct4 and Nanog promotes tumorigenic and metastatic abilities in nude mice Xenograft tumor models.

### Oct4 functions with Nanog in regulating EMT by activation of Stat3 pathway

**Figure 4:**
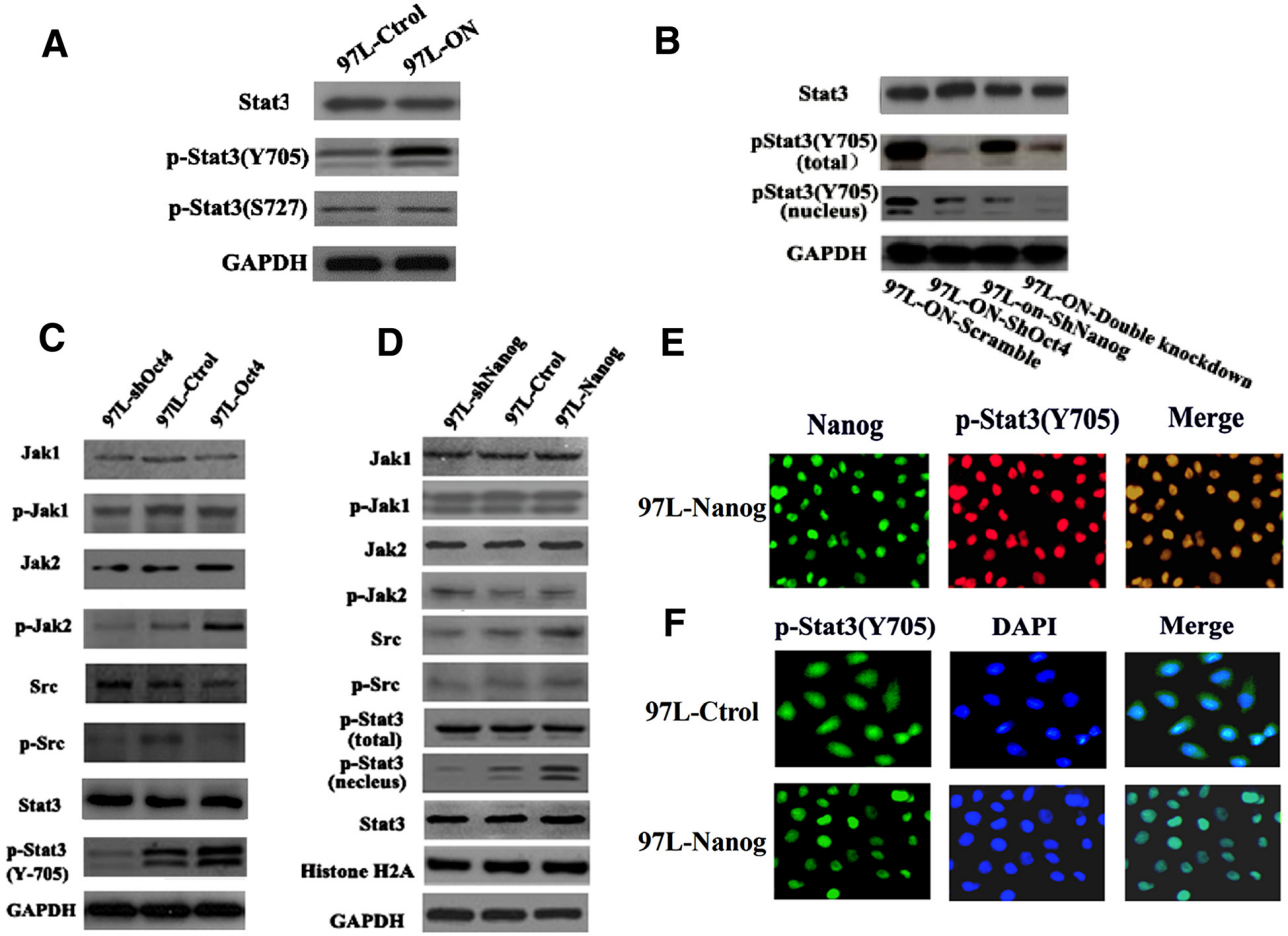
**Oct4 /Nanog regulates EMT by activating Stat3 pathway. (A)** Stat3, p-Stat3 (Y705), and p-Stat3 (S727) protein expression in 97 L-Ctrol cells and 97 L-ON cells. 97 L-ON cells expressed significantly higher amounts of stat3 and p-Stat3 (Y705) than 97 L-Ctrl cell lines. **(B)** Stat3, p-Stat3^Y-705^ (total protein and nucleus protein) expression in 97 L-ON-Scramble, 97 L-ON-shOct4, 97 L-ON-shNanog, 97 L-ON-double knockdown cells. **(C)** Expression of Stat3, p-Stat3 (Y705) proteins, Jak1, p-Jak1, Jak2, p-Jak2 in 97 L-Ctrol, 97 L-shOct4, and 97 L-Oct4 cells. **(D)** Effect of Nanog expression on stat3 expression, activation, and translocation. Western blot analysis revealed that Nanog had no effect on Stat3 protein, total p-Stat3 (Y705), and p-Jak1, p-Jak2, p-Scr protein expression but increased p-Stat3 (Y705) amounts in nucleus, which indicated nuclear translocation of phospho-Stat3 induced by Nanog. **(E)** Immunostaining of Nanog (Alexa Fluor®488-labeled anti-Nanog, green color) and p-Stat3 (Alexa Fluor®568-labeled anti-Nanog, red color). Colocalization of Nanog and p-Stat3 (Y-705) is shown as yellow in the merged image. **(F)** Overexpression of Nanog promoted p-Stat3 nuclear translocation in immunofluorescence analysis. p-Stat3 (Y-705) was localized in cytoplasm and nucleus in 97 L-Ctral cells. After overexpression of Nanog, staining of p-Stat3 protein (green) was significantly increased in the nucleus of 97 L-Nanog cells.

Previously, we have observed that 97 L-ON cells contained a mesenchymal-like phenotype, whereas the 97 L-Ctrol cells stayed in their original epithelial-like morphology. The regulation of EMT involves complex signaling pathways mainly including TGF-β, Wnt/β-catenin, Stat3, Notch, and Hedgehog pathway [14], leading to enhanced tumor invasion and metastasis. It has been reported that Oct4 can regulate signal transducer and activator of transcription 3 (Stat3) expression in embryonic stem cells [15], while a functional cooperation between Nanog and p-Stat3 translocation was also implied in breast and ovarian cancer [16]. We asked whether Oct4 functions with Nanog in regulating EMT by activation of Stat3 pathway. We examined the expression levels of Stat3, p-Stat3 (Y705), and p-Stat3 (S727) expression in 97 L-ON cells and 97 L-Ctrol cells (Figure 4A). The results showed that 97 L-ON cells expressed higher amounts of p-Stat3 (Y705) than 97 L-Ctrol cells.

To test the independent role of Oct4 or Nanog in Stat3 activation in 97 L-ON cells, we treated the 97 L-ON cells with either Nanog shRNA and/or OCT4 shRNA and examined the effect of Oct4/Nanog knockdown on Stat3 pathway *in vitro*. As shown in Figure 4B, knockdown expression of Oct4 significantly reduced the expression of total tyrosine phosphorylated Stat3 (p-Stat3^Y705^). While knockdown Nanog had no effect on total p-stat3, it reduced p-stat3 in nucleus (p-Stat3^Y705^). Double-knockdown Oct4/Nanog significantly decreased p-Stat3 protein in cytoplasm and nucleus. This finding manifested that Oct4 might upregulate the total p-Stat3 (Y705) protein level in HCC and Nanog might regulate p-Stat3 nuclear localization.

To further investigate the role of Oct4 in regulation of Stat3, we established 97 L-shOct4 cells transfected with Oct4 shRNA to downregulate endogenous Oct4 expression and 97 L-Oct4 transfected with plasmid to upregulate Oct4, with empty plasmid as control. As expected (Figure 4C), silencing of Oct4 induced a reduction in p-Stat3 (Y705) protein, whereas overexpression of Oct4 increased the expression of p-Stat3 (Y705). To elucidate the exact mechanism by which Oct4 regulates p-Stat3, we examined Stat3 upstream kinase molecules Jak1, p-Jak1, Jak2, p-Jak2, Scr, and p-Scr. The results showed that overexpression of Oct4 augmented the expression of Stat3 upstream kinases p-Jak2 expression. Then, we treated 97 L-Oct4 cells with AZD1480 (AstraZeneca, Waltham, MA, USA), a Jak2-specific inhibitor which blocks Stat3 activation and its downstream signaling. Western blot analysis showed inhibition of Jak2 abrogated Oct4-induced phosphorylation of Stat3 and its downstream genes CyclinD1 and Survivin (Additional file 1: Figure S1). These results clearly indicated that Oct4 mediates Stat3 activation through Jak2 pathway.

We then assessed the effect of Nanog expression on Stat3 activation and translocation. 97 L-sh Nanog cells transfected with Nanog shRNA to downregulate endogenous Nanog expression and 97 L-Nanog transfected with plasmid to upregulate Nanog were established. As expected, we found an increase of p-Stat3 in the nucleus of 97 L-Nanog cells and a decrease of p-Stat3 in the nucleus of 97 L-sh Nanog cells. No significant changes were found in total p-Stat3 protein and its upstream kinases Jak1, p-Jak1, Jak2, p-Jak2, Src, and p-Src expression (Figure 4D). Then, we used immunofluorescence to assess the co-location of Nanog and p-Stat3 (Y705) in 97 L-Nanog cells. As showed in Figure 4E, both red fluorescence and green fluorescence were accumulated at the nucleus of cell clusters and overlapped as yellow fluorescence, indicating the co-location of Nanog and p-Stat3. Figure 4F showed that in 97 L-Ctrol cells, p-Stat3 was predominantly found both in cytoplasm and nucleus, whereas it appeared to be primarily retained within the nucleus in 97 L-Nanog cells, which indicated that overexpression of Nanog in 97 L cells promoted p-Stat3 (Y705) translocation into the nucleus.

**Figure 5:**
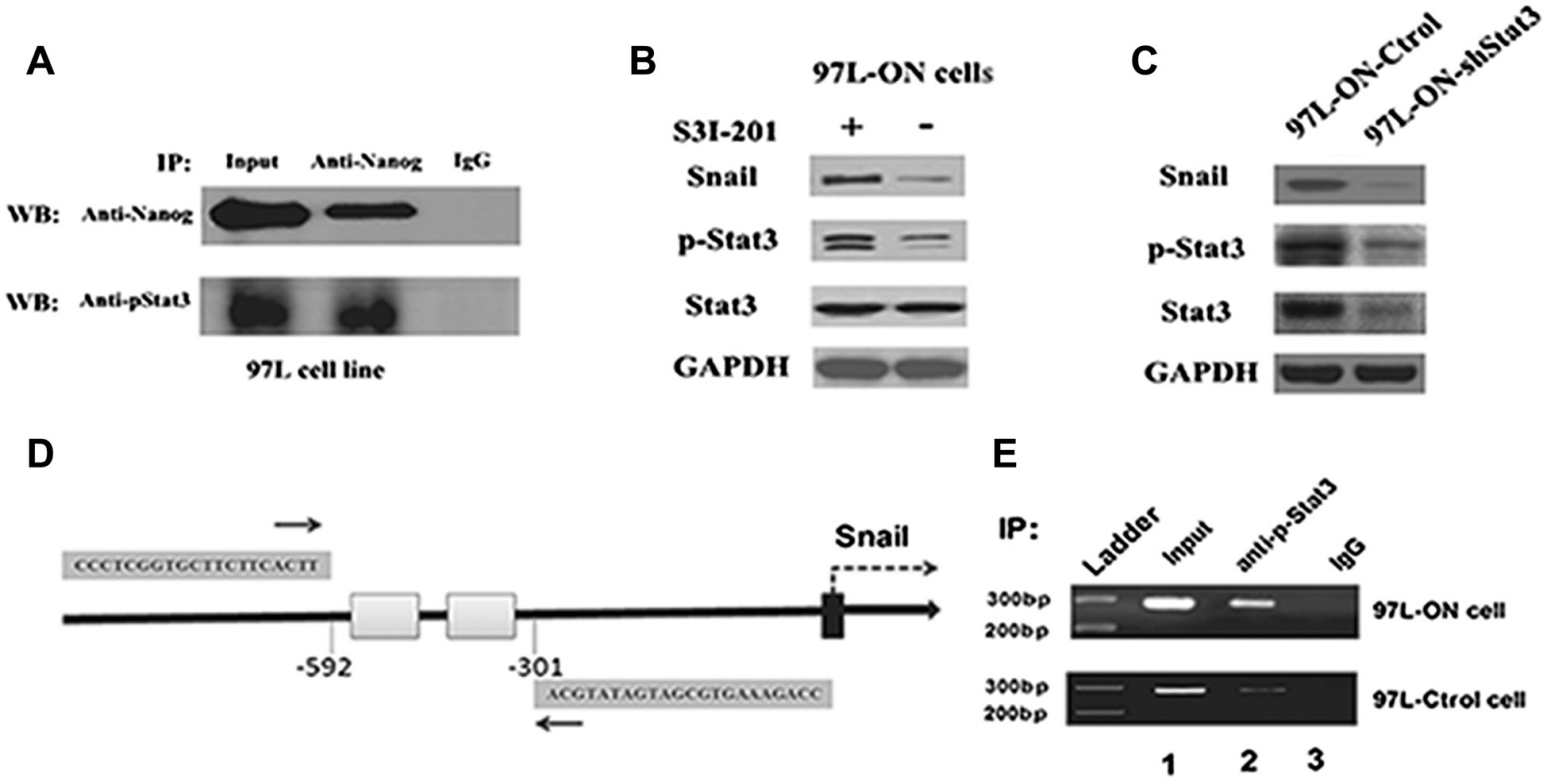
**Oct4/Nanog-mediated Stat3 activation regulates snail expression in 97 L-ON cells. (A)** Association of Nanog with p-Stat3 in the nucleus of 97 L cell line. Equal amounts of protein were immunoprecipitated (IP) with an anti-Nanog monoclonal antibody or anti-p-Stat3-Y705 antibody and were immunoblotted to detect Nanog or p-Stat3 (Y-705). Normal mouse IgG was used as a control antibody. **(B)** Oct4/Nanog-induced Snail expression was significantly inhibited by a specific Stat3 inhibitor S3I-201 (10 μM for 48 h). **(C)** Knockdown of Stat3 reversed Oct-4/Nanog-induced overexpression of Snail in 97 L-ON cells. **(D)** Schematic representation of structure of Snail promoter reign. Grey rectangle represented putative Klf4 binding sites predicted using MatInspector. Black arrows (thin) depicted the location of the forward and reverse primers used for PCR amplification from immunoprecipitated DNA fragments. **(E)** Chromatin immunoprecipitation (ChIP) analysis of p-Stat3 enrichment in Snail promoter. A p-Stat3 antibody or IgG serum was conducted to immunoprecipitate DNA-protein complexes from 97 L-ON cells with p-Stat3 overexpression. IgG was used for a negative control. Binding of p-Stat3-containing transcription complex on the *Snail* promoter was enriched in 97 L-ON cells, compared with 97 L-Ctrol cell.

In order to examine whether Nanog interacts with p-Stat3 (Y705) directly in the nucleus of HCC cells, we analyzed the anti-Nanog-mediated immunoprecipitates from nuclear extracts by immunoblotting with anti-p-Stat3 (Y705), or anti-Nanog antibody, respectively, in 97 L cell lines. Our results demonstrated a measurable amount of Nanog, p-Stat3 complexes (Figure 5A) in the nuclear fractions of 97 L cell lines. Furthermore, the same results were also confirmed in another HCC cell lines (HepG2 cell lines, Additional file 2: Figure S2). These findings suggested that Nanog is capable of forming a complex with p-Stat3 (Y705) in cell line in an independent manner.

### Oct4/Nanog-mediated Stat3 activation regulates snail expression in 97 L-ON cells

Our previous experimental study indicated that overexpression of Oct4/Nanog significantly increased the expression of Snail, but not Slug or Twist, at both mRNA and protein levels in 97 L-ON cells (Figure 1B). It has also been reported that the activation of Stat3 induced EMT through Snail activation in head and neck tumor [17]. To determine whether Oct4/Nanog-promoted Snail expression is mediated by Stat3 phosphorylation, we treated 97 L-ON cells with S31-201 [18], a specific Stat3 inhibitor, effectively inhibited Stat3 phosphorylation, dimerization, and translocation to nucleus. As showed in Figure 5B, Oct4/Nanog-induced Snail expression was significantly inhibited by S3I-201. To further confirm these results, we examined the effects of shRNA-mediated Stat3 knockdown on Snail expression. Indeed, knockdown of Stat3 dampened Oct-4/Nanog-induced expression of Snail expression in 97 L-ON cells (Figure 5C).

Since knockdown of Stat3 expression greatly reduced snail mRNA levels, we assessed whether Stat3 inhibited the activity of the Snail gene promoter by chromatin immunoprecipitation (ChIP) assay. p-Stat3 antibody or IgG serum was conducted to immunoprecipitate DNA-protein complexes from 97 L-ON cells in which Stat3 is constitutively active. According to bioinformatic prediction, there are two Stat3 consensus binding sites in the mouse Snail promoter (from −592 to −301 bp, Figure 5D). Compared with 97 L-Ctrol cell, p-Stat3 binding on the Snail promoter were significant enriched in 97 L-ON cell (Figure 5E). These results showed that Stat3 activation is involved in Oct4/Nanog regulation on Snail expression.

### Silencing Stat3 abrogates Oct4/Nanog-mediated EMT changes and invasion/metastasis of HCC

**Figure 6:**
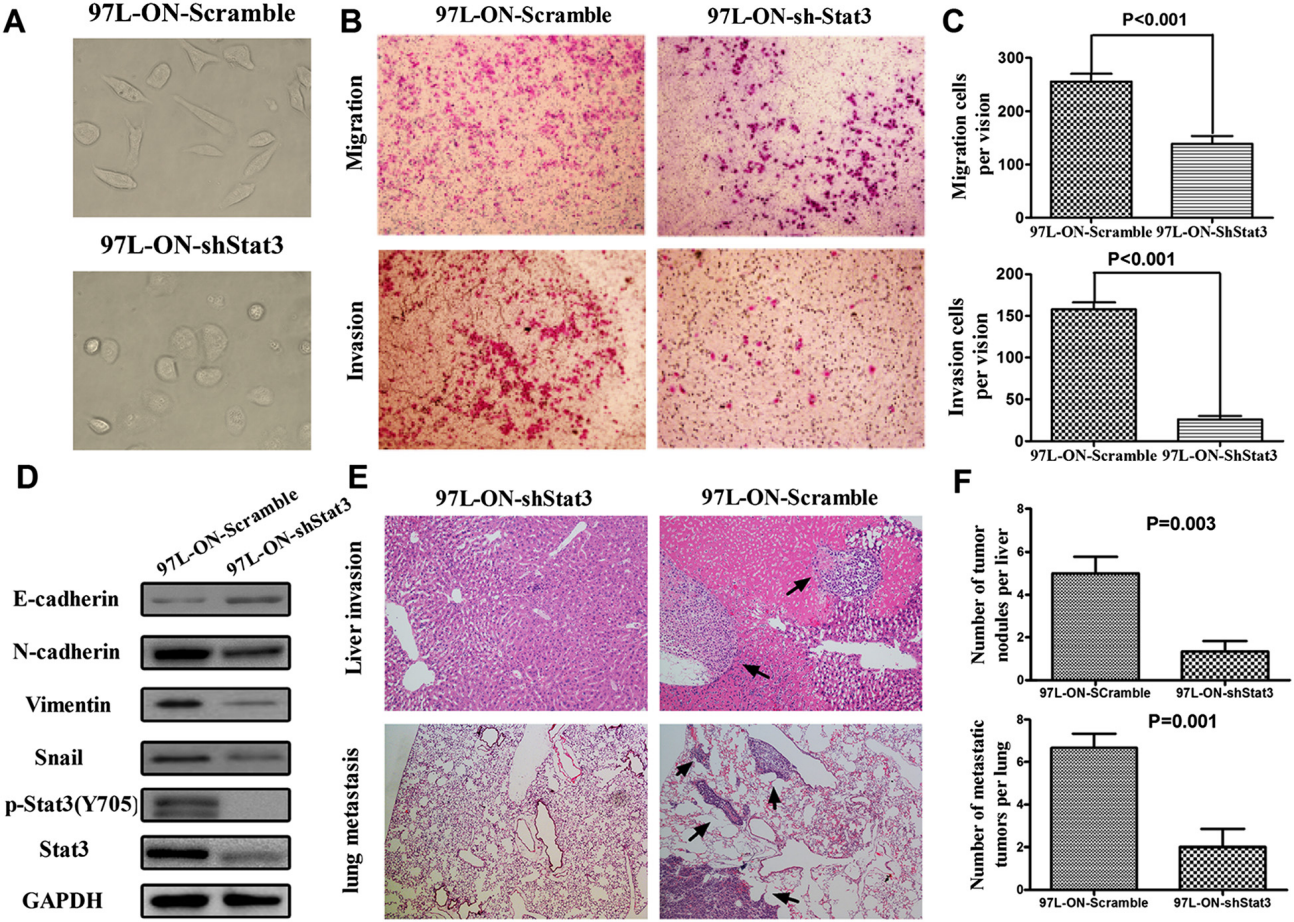
**Silencing Stat3 dampens EMT phenotype and attenuates invasion/metastatic ability of 97 L-ON cells in vitro and vivo. (A)** 97 L-ON, mesenchymal, fibroblast-like cancer cells underwent morphologic change into epithelial phenotype after knockdown Stat3. **(B)** Representative photographs of cell migration and invasion. **(C)** Quantification migration and invasion assay indicated that the numbers of migrated or invaded 97 L-ON-shStat3 cells were less than those of 97 L-ON-Scramble cells (*P* < 0.001). **(D)** Significant decreases in the mesenchymal genes, N-cadherin, Snail, and Vimentin and increase in the epithelial gene E-Cadherin were found in 97 L-ON-shStat3 cells by Western blot analysis. **(E)** Up panel, representative photographs of intrahepatic invasion (H & E staining, black arrows point to the invasive nodules, ×100 magnification). Bottom panel, representative photographs of lung metastasis (H & E staining, black arrows point to the metastatic nodules, ×40 magnification). **(F)** Quantification analysis showed the numbers of liver dissemination (up panel) and lung metastasis (bottom panel) of 97 L-ON-shStat3 cells were both less than those of 97 L-ON-Scramble cells (*P* = 0.003 and *P* = 0.001).

Because Stat3 was correlated with Oct4/Nanog-mediated EMT, we investigated the impact of Stat3 knockdown in EMT changes and invasion/metastasis of 97 L-ON cells. We found that after silencing Stat3, 97 L-ON cell underwent morphologic change, from mesenchymal phenotype to epithelial phenotype (Figure 6A). Accompanied with morphologic change, significant decreases in the mesenchymal genes, N-cadherin, Snail, and Vimentin and increase in the epithelial gene E-Cadherin were found in 97 L-ON-shStat3 cells in Western blot analysis (Figure 6D). Furthermore, the numbers of migration and invasion of 97 L-ON-ShStat3 cells were significantly lower than 97 L-ON-Scramble cells (Figure 6B, C). Then, we investigated the effects of Stat3 knockdown on liver dissemination and lung metastasis of HCC cells *in vivo*. Consistent with those *in vitro* findings, 97 L-ON-shStat3 knockdown xenograft tumors displayed less liver dissemination and lung metastasis in nude mice compared with 97 L-ON-Scramble tumors (Figure 6E, F). All these findings demonstrated that silencing Stat3 expression abrogated Oct4/Nanog-mediated EMT change and invasion/metastasis of HCC.

## Discussion

Previously, we have demonstrated that Oct4 and Nanog are coexpressed and significantly upregulated in HCC patients with early recurrence/metastasis and poor outcomes [13]. In the present study, we established ectopic coexpression of Oct4 and Nanog 97 L-ON cell lines. We found that 97 L-ON cells exhibited typical stem-like properties, such as sphere formation ability, anchorage-independent growth, chemotherapy resistance, and high tumorigenicity. More strikingly, ectopic coexpression of Oct4 and Nanog encouraged EMT in HCC, promoted migration and invasion during HCC metastasis. Further molecular mechanism revealed that Oct4/Nanog targeted Stat3 pathway in HCC and regulated the expression and function of Snail, thus promoted EMT in HCC.

Self-renewal and chemoresistance are two important characteristics of CSCs. To test whether Oct4/Nanog initiate self-renewal properties in HCC, we performed colony formation ability assay and sphere-forming ability assay *in vitro* and tumorigenecity assay *in vivo*. Our study demonstrated that overexpression of Oct4 and Nanog significantly promoted self-renewal capacity of 97 L HCC cell lines (Figure 1 and Table 1). Specifically, coexpression of Oct4 and Nanog increased the expression of stem cell markers CD133, ALDH1, and BMi-1. In addition, exogenous Oct4 and Nanog expression substantially enhanced the expression of ABCG2 and MDR1, two major ABC transporters which attribute to drug resistance. To further assess whether 97 L-ON cells possess a hypothesized CSC chemoresistant property, we examined the sensitivity of 97 L-ON cells to chemotherapeutics. The results suggested that 97 L-ON cells exhibited general resistance to DDP, compared with its control cells. Although the underlying molecular mechanism of Oct4 and Nanog on CSCs drug resistance is unclear, it manifested that stem cell genes activation are responsible for the failure of eradicating cancer cells and preventing tumor recurrence in current chemoradiation therapy.

The expression of Oct4 or Nanog has been reported in the cancer stem-like cells and associated with a more primitive and aggressive tumor phenotype [19]. Consistent with previous studies, we found that ectopic overexpression of Oct4 and Nanog converted 97 L cellular morphology to a fibroblast-like morphology (EMT change). Further cell proliferation assay, wound healing, and transwell assay indicated that Oct4/Nanog increased 97 L cell proliferation, invasion, and metastasis *in vitro*. More importantly, *in vivo* xenograft tumorigenicity, tumor invasion, and metastasis assays confirmed that Oct4/Nanog contributed to HCC intrahepatic dissemination and lung metastasis. Therefore, it is conceivable that the core transcriptional regulatory factor Oct4 and Nanog played an important role in promoting tumorigenesis, invasion/metastasis in HCC.

In the present study, we identified that Stat3 activation played a mechanistic role in Oct4/Nanog-induced EMT and cell invasion in HCC. Actually, biological function between Oct4, Nanog, and Stat3 has been explored in previous study. In embryonic stem cells, it has been shown that Oct4 is essential for antiapoptotic effects in response to stress, and these effects may be mediated through the activation of Stat3 pathway [15]. More interestingly, Nanog can form a complex with the p-Stat3, leading to Stat3-specific transcriptional activation in breast and ovarian tumor cells [16]. Using Oct4/Nanog overexpressing, as well as knockdown in HCC cell lines, we showed that in HCC, Oct4 mediated Stat3 activation while Nanog mediated p-stat3 nuclear translocation and binding to the Snail promoter. Our findings suggested that targeting Oct4/Nanog-mediated Stat3 signaling pathway may represent a novel approach to overcome EMT process in liver cancer cells displaying stem cell marker properties during tumor progression.

Snail is one of the best-characterized E-cadherin gene repressors required for triggering EMT. It represses E-cadherin transcription by binding to the E-box site in the promoter of E-cadherin [20]. In addition to its role in the repression of E-cadherin, Snail is also known to stimulate mesenchymal gene transcription such as N-cadherin and Vimentin expressions [21]. We found that Oct4/Nanog-mediated Stat3 activation is highly important for Snail expression in HCC. The mRNA and protein levels of Snail were increased upon Oct4/Nanog-mediated Stat3 activation and significantly decreased by the inhibition or knockdown of Stat3 expression (Figure 5B, C). Results of the ChIP assay (Figure 5E) further supported the regulatory role of Oct4/Nanog/Stat3 signaling on Snail promoter. This result is consistent with another report, which showed that Snail expression is regulated by Stat3 signaling pathway in breast cancer during epithelial mesenchymal transition [22]. It provides a mechanistic explanation for the prognostic studies that have directly linked Oct4/Nanog [13], Stat3 signaling [23], and Snail [24] with tumor recurrence, tumor metastasis, and poor survival in HCC patients.

Increasing evidence shows a direct link between the EMT and CSCs. Both EMT and CSC are implicated in the generation of invasive cells and formation of distant metastases. Furthermore, CSCs have been found to express EMT-associated genes in addition to stemness-associated genes. However, there is still a lack of evidence to explain the biological similarities between CSCs and EMT-phenotypic cancer cells. To the best of our knowledge, this is the first report to illustrate how stemness transcription factor Oct4 and Nanog regulates EMT signaling in HCC.

Our study indicates a potential link between Oct4/Nanog and Stat3/Snail signaling. This model of signaling regulation between CSC characteristics and EMT pathway may partially explain how cancer stem cells maintain their aggressiveness in the long period of time when invading and migrating to surrounding tissues.

Taken together, our results provide novel insight into the role of stem cell genes Oct4/Nanog in promoting CSC-like traits and EMT change in HCC. As Oct4/Nanog is actively involved in EMT process via regulation on Stat3/Snail pathway, we postulate that inhibition of Stat3 pathway would be a promising therapeutic strategy to control stem cells-associated EMT phenotype in HCC.

## Methods

### Cell culture and transfection

MHCC97-L (97 L), a low-metastatic human HCC, was established at our institution [25]. HepG2 cells were purchased from the Shanghai cell bank, Chinese Academy of Sciences. Human Oct4, and Nanog genes were PCR amplified from normal genomic DNA and cloned into lentiviral vector pGMLV-PA6 (Genomeditech Co., Ltd., Shanghai, China) for ectopic expression of Oct4 and Nanog. Primers used for Oct4 and Nanog amplification were listed in Additional file 3: Table S1. Oct4, Nanog, or mock vectors were transfected into 97 L cells using Lipofectamine 2000 reagent (Invitrogen, Carlsbad, CA, USA). The transfected cells were selected under 3 μg/mL puromycin (P8833, Sigma-Aldrich, St. Louis, MO, USA). Clones of stably transected cells were obtained by the limited dilution method. Cells with coexpression of Oct4 and Nanog were named as 97 L-ON cells and the expression of Oct4 and Nanog was confirmed by qRT-PCR and Western blot analysis.

To generate plasmids that express specific short hairpin RNA, three sequences of shRNA targeting Oct4, Nanog, and Stat3 were designed and cloned into cloning vector pGMLV-SC1 (Genomeditech Co., Ltd.). The best knockdown shRNA sequences were listed in Additional file 3: Table S1. The sequence of Scrambled RNA was used to generate the negative control. HCC cells transfected with the lentiviral particles were selected with 3 μg/mL puromycin.

### Real-time PCR and Western blot

Real-time RT-PCR procedures were performed according to the manufacturer’s instructions. The primers for amplification of human genes were present in Additional file 3: Table S1. Western blots were performed as described previously [26]. Abs against Oct4 (Cat. #ab19857) and Snail (Cat. #ab180714) were brought from Abcam (AbCam, Cambridge, UK). Abs against Nanog, total Stat3 (Cat. #9139), p-Stat3Tyr705 (Cat. #9145), p-Stat3Ser727 (Cat. #9130), Jak1 (Cat. #3332), p-Jak1 (Cat. #3331), Jak2 (Catalog: #3230), p-Jak2 (Cat. #3771), Src (Cat. #2108), p-Src (Cat. #2105), and Histone H2A (Cat. #2578) were brought form Cell Signaling Technology (Cell Signaling Technology, Danvers, MA, USA). Abs against E-cadherin (Cat. # sc-7870), N-cadherin (Cat. #sc-7989), Slug (Cat. #sc-15391), Twist (Cat. #sc-15393), and Vimentin (Cat. # sc-5565) were purchased from Santa Cruz Biotechnology (Santa Cruz Biotechnology, CA, USA). Ab against GAPDH (Cat. # KC-5G4) was from Kangcheng (Kangcheng Technology, Shanghai, China).

### Cell proliferation assay

The measurement of cell proliferation was performed with Cell Counting Kit-8 (Dojin Laboratories, Kumamoto, Japan). Cells (2 × 10^3^ cells/well) were seeded in 96-well plates and continuously incubated for 2 h with Cell Counting Kit-8 solution. The absorbance of sample taken from each well was measured on a spectrophotometer (Thermo Electron, Andover, USA) at 450 nm. The results were plotted as the mean ± SD from three separate experiments with six replicates per experiment for each experimental condition.

### Colony formation assay

Cells were plated at a concentration of 1.5 × 10^3^ cells in 60-mm^2^ culture dishes and cultured for 7 days, then fixed with methanol and stained with 0.5% crystal violet for 15 min. The dishes were photographed, and the colonies were counted.

### Spheroid body-forming assay

Cells were counted and seeded at 2,000 cells/well in a 96 well ultra-low-attachment plate (Corning Incorporated, Corning, NY, USA) and cultured with DMEM-F12 medium supplemented with 2% B-27 supplement (Invitrogen), 20 ng/ml human bFGF, and 20 ng/ml EGF (Chemicon, Temecula, CA, USA). Once spheres began to form, they were quantified using inverted contrast microscopy (Leica DMI 3000B, Wetzlar, German) by counting the number of spheres per culture well.

### Wound healing assay

Cells were cultured for 2 days to form a tight cell monolayer and then serum-starved for 16 h. After the serum starvation, the cell monolayer was wounded with a 10 μl plastic pipette tip. At the indicated times, migrating cells at the wound front were photographed. A percentage of the wound area at each time point was measured using Image-Pro Plus v6.2 software.

### Transwell assay

The migration or invasion assays were carried out using transwell chamber with 6.5-mm diameter polycarbonate filters (8 μm pore size, BD Biosciences, Erembodegem, Belgium) coated with or without matrigel. Cells (5 × 10^4^) in serum-free media were seeded in the top chamber and 20% FBS was used as the chemoattractant in the bottom chamber. After incubation for 24 h, all of the non-invaded (or non-migrated) cells were removed, and cells that have invaded (or migrated) through the membranes were fixed with methanol, stained with Giemsa stain. Quantification of the invaded (or migrated) cells was done by counting four regions of the filter under the microscope. Triplicate filters were used and the experiments were repeated three times.

### *In vivo* xenograft tumorigenicity, tumor invasion, and metastasis assays

Male athymic BALB/c nude mice were purchased from Shanghai Institute of Material Medicine, Chinese Academy of Science, and were raised in specific pathogen-free conditions. Animal care and experimental protocols were conducted in accordance with guidelines established by the Shanghai Medical Experimental Animal Care Commission.

For *in vivo* tumorigenicity experiments, 97 L-ON cells or 97 L-Ctrol cells (5 × 10^3^, 5 × 10^4^, 5 × 10^5^, respectively) were injected subcutaneously into the right flank of each mouse (six mice per group). The mice were observed for tumor growth every day over 6 to 8 weeks and then sacrificed by cervical dislocation.

For *in vivo* tumor invasion and lung metastasis assays, 5 × 10^6^ 97 L-ON Cells and 97 L-Ctrl cells were subcutaneously inoculated into the right flanks of the nude mice. After 6 weeks, subcutaneous tumors were surgically excised, weighed, and photographed. Nonnecrotic tumor tissue was cut into 1-mm^3^ pieces and orthotopically implanted into the liver. Intrahepatic dissemination and lung metastasis were determined by pathological examinations.

### Immunofluorescence staining

Briefly, cells cultured on glass slides were fixed by 4% paraformaldehyde. The slides were incubated with primary antibodies against Nanog, p-Stat3Tyr705 for 1 h and followed by the secondary antibodies Alexa Fluor®488 goat anti-rabbit and Alexa Fluor®568 goat anti-rabbit IgG (1:800 dilution; Invitrogen) for 1 h. Nuclei were stained with DAPI (Invitrogen). Fluorescent images were visualized using a confocal laser scanning microscope (FV-1000; Olympus Tokyo, Japan).

### Co-immunoprecipitation analysis

Immunoprecipitation was performed with 97 L cells and HepG2 cells. The interaction between Nanog and p-Stat3 was analyzed. Cells were plated in 100-mm^2^ dishes (1.5 × 10^6^/dish) and were harvested with lysis buffer. A quantity of 4 mg of protein was mixed with 40 μl of Protein A-Sepharose beads (Sigma) in the immunoprecipitation assay buffer, incubated at 4°C for 2 h with gentle agitation and centrifuged for 10 min at 2,000 rpm for preclearing. The recovered supernatant was incubated with 2 μg of anti-Nanog (1:1,000) or anti-phospho-Stat3 Try705 antibody (1:1,000) in the presence of protease inhibitors at 4°C overnight. Then 50 μl of Protein A-Sepharose beads was added, and the incubation was continued for 2 h at 4°C with gentle shaking. The Protein A-precipitated protein complex was recovered by a brief centrifugation followed by three washes with the immunoprecipitation assay buffer. The harvested beads were resuspended in 30 μl of 2 × SDS PAGE sample buffer and boiled for 5 min to release the bound protein. A 20 μg aliquot of cell lysate was used as an input control. The samples were analyzed by Western blot.

### Chromatin immunoprecipitation

Chromatin immunoprecipitation assay was performed using a Chromatin Immunoprecipitation Assay Kit (Millipore, Billerica, MA, USA) according to the manufacturer’s instructions. Immunoprecipitations were preformed with anti-pStat3 or mouse IgG (Cell Signaling Technology) as a negative control. Immunoprecipitated DNA was analyzed by real-time PCR with SYBR Green. The resulting PCR product which spans the −592 to −301 region of Snail promoter includes the putative Stat3-binding sites. Specific sequences of the human Snail promoter in the immunoprecipitates were detected by PCR with primers forward 5′-CCCTCGGTGCTTC TTCACTT-3′ and reverse 5′-CCAGAAAGTGCGATGATATGCA-3′.

### Statistical analyses

All experiments were repeated at least three times and representative results are presented. All values in the figures and text are the means ± SD. Statistical analyses were performed using the SPSS 13.0 for Windows (SPSS, Inc., Chicago, IL, USA). Any significant differences among mean values were evaluated by Student *t* test or Mann–Whitney *U* test. A two-sided *P* < 0.05 was accepted as significant.

## Additional files

Additional file 1: Figure S1. Inhibition of Jak2 activation in 97 L-Oct4 cells by Jak2 inhibitor AZD1480. 97 L-Oct4 cells were treated with 0, 1.0, 2.5, 5, 10 μmol/L AZD1480 and, respectively, for 48 h. Inhibition of p-Jak2 by AZD1480 abrogated Oct4-induced phosphorylation of Stat3 and its downstream genes CyclinD1 and Survivin. Additional file 2: Figure S2. Immunoprecipitation assay confirmed Nanog interacts with p-Stat3 (Y-705) in the nucleus of HepG2 cell line. Equal amounts of protein were immunoprecipitated (IP) with an anti-Nanog monoclonal antibody or anti-p-Stat3-Y705 antibody and were immunoblotted to detect Nanog or p-Stat3 (Y-705). Normal mouse IgG was used as a control antibody. Additional file 3: Table S1. Sequences for and shRNAs and primers for gene amplification in this study.

## Abbreviations

ALDH1: aldehyde dehydrogenase 1
CSC: cancer stem cells
DDP: cisplatin
EMT: epithelial-mesenchymal transition
HCC: hepatocellular carcinoma
Stat3: signal transducer and activator of transcription 3
