# Supplementary figures and images for: Coexpression of gene Oct4 and Nanog initiates stem cell characteristics in hepatocellular carcinoma and promotes epithelial-mesenchymal transition through activation of Stat3/Snail signaling
Source: J Hematol Oncol. 2015 Mar 11;8:23. doi: 10.1186/s13045-015-0119-3 (PMC4377043; doi:10.1186/s13045-015-0119-3)

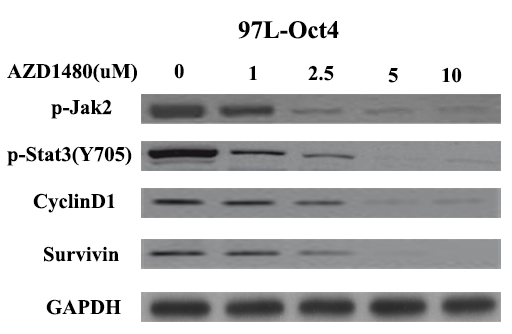

Supplement: Additional file 1: Figure S1. — Inhibition of Jak2 activation in 97 L-Oct4 cells by Jak2 inhibitor AZD1480. 97 L-Oct4 cells were treated with 0, 1.0, 2.5, 5, 10 μmol/L AZD1480 and, respectively, for 48 h. Inhibition of p-Jak2 by AZD1480 abrogated Oct4-induced phosphorylation of Stat3 and its downstream genes CyclinD1 and Survivin. [file 13045_2015_119_MOESM1_ESM.tiff]

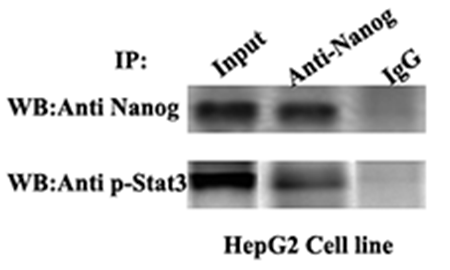

Supplement: Additional file 2: Figure S2. — Immunoprecipitation assay confirmed Nanog interacts with p-Stat3 (Y-705) in the nucleus of HepG2 cell line. Equal amounts of protein were immunoprecipitated (IP) with an anti-Nanog monoclonal antibody or anti-p-Stat3-Y705 antibody and were immunoblotted to detect Nanog or p-Stat3 (Y-705). Normal mouse IgG was used as a control antibody. [file 13045_2015_119_MOESM2_ESM.tiff]
