# Supplementary material for: Coexpression of gene Oct4 and Nanog initiates stem cell characteristics in hepatocellular carcinoma and promotes epithelial-mesenchymal transition through activation of Stat3/Snail signaling
Source: J Hematol Oncol. 2015 Mar 11;8:23. doi: 10.1186/s13045-015-0119-3 (PMC4377043; doi:10.1186/s13045-015-0119-3)
Supplement: Additional file 3: Table S1. — Sequences for and shRNAs and primers for gene amplification in this study. [file 13045_2015_119_MOESM3_ESM.doc]

**Supplementary Table S1**

| **Type** | **Sense sequence** | **Anti-sense sequence** |
| --- | --- | --- |
|  |  |  |
| **Oct4** | GATCCGGTTCTATTTGGGAAGGTATTAGAGAACTTAATACCTTCCCAAATAGAACCTTTTTTG | AATTCAAAAAAGGTTCTATTTGGGAAGGTATTAAGTTCTCTAATACCTTCCCAAATAGAACCG |
| **Nanog** | GATCCGCATGCAGTTCCAGCCAAATTAGAGAACTTAATTTGGCTGGAACTGCATGCTTTTTTG | AATTCAAAAAAGCATGCAGTTCCAGCCAAATTAAGTTCTCTAATTTGGCTGGAACTGCATGCG |
| **Stat3** | GCAGCAGCTGAACAACATGCATGTTCAAGAGACATGTTGTTCAGCTGCTGCTTTTT | AATTAAAAAGCAGCAGCTGAACAACATGTCTCTTGAACATGTTGTTCAG CTGCTGCTGCGGCC |
| **Primer for gene amplification** | |  |
| **Oct4** | CCGGAATTC GCCACCATGGCGGGACACCTGGCT | CGGGATCCTCACTTGTCGTCATCGTCCTTGTAGTCGTTTGAATGCATGGGAGAGCC |
| **Nanog** | GAGGATCCCCGGGTACCGGTCGCCAC | TCACCATGGTGGCGACCGGCACGTCTTCAGGTTGCATGT |
| **CD133**  **Bmi-1**  **CD44**  **ALDH1**  **EPCAM**  **MDR1**  **ABCG2**  **E-cadherin**  **N-cadherin** | TTTCAAGGACTTGCGAACTCTC  CAACTGGTTCGACCTTTGCAGATA  TTGTGGCATTTATTCATCAG  TCCAGCCCACAGTGTTCTCTAAT  CAGTTGGTGCACAAAATACTGTCA  GAGGAAGACATGACCAGGTA  GGATGAGCCTACAACTGGCTT  TTAAACTCCTGGCCTCAAGCAATC  CACCCAACATGTTTACAATCAACAATGAGAC  CCTGGGTGCCCTCAAGAT  AGTTCGTAAAGGAGCCGGGTGA  GTCCGCAGTCTTACGAGGAG  TGAGTACCGGAGACAGGTGCAG  AGCCACATCGCTCAGACA | TGCTACAGCTGGCTTAGAGAC  GATGTGCCAATTGCTTCTAATGGA  GGTAGACAGGGAGGAGCA  GATTTGCTGCACTGGTCCAA  CCATTCATTTCTGCCTTCATCA  CTGTCGCATTATAGCATGAA  TTCCTGAGGCCAATAAGGTG  TCCTATCTTGGGCAAAGCAACTG  CTGCAGCAACAGTAAGGACAAACATCCTATT |
| **Snail**  **Slug**  **Twist**  **Vimentin**  **GAPDH** |  | GTGGAGCAGGGACATTCG  TTGGGTAGCTGGGCGTGGA  GCTTGAGGGTCTGAATCTTGCT  TAGCAGCTTCAACGGCAAAGTTC  GCCCAATACGACCAAATCC |

**Sequences for and shRNAs and primers for gene amplification in this study**
